# Beyond responsiveness: Differentiated associations among product features, psychological needs, perceived enjoyment, and continuance intention toward AI learning devices

**DOI:** 10.1371/journal.pone.0358228

**Published:** 2026-09-21

**Authors:** Gang Ren, Xuezhen Wu, Gang Wang, Yinuo Ye, Zhihuang Huang, Tianyang Huang

**Affiliations:** 1 School of Design Arts, Xiamen University of Technology, Xiamen, China; 2 Sierra Canyon School, Chatsworth, California, United States of America; Zhengzhou University, CHINA

## Abstract

As large language models are increasingly embedded in dedicated learning tablets, educational robots, and other physical AI learning devices (AILDs), sustaining learners’ engagement beyond initial adoption has become a broader challenge for educational technology research and design. Although the technical capabilities of such devices continue to advance, the factors associated with continued use remain poorly understood. Drawing on the Stimulus–Organism–Response framework and Self-Determination Theory, this study examines associations among perceived personalization, interactivity, response timeliness, friendliness, autonomy, competence, relatedness, perceived enjoyment, and continuance intention. Cross-sectional survey data were collected from 726 first-year senior high school students with experience using AILDs and analyzed using partial least squares structural equation modeling. Effect sizes varied substantially across the estimated associations. The association between relatedness and perceived enjoyment had the largest effect size (*f*^2^ = 0.266), whereas the remaining effect sizes were small or negligible. Two theoretically informative expected associations were not supported: perceived personalization with autonomy and response timeliness with relatedness. The model explained 47.0% of the variance in continuance intention and 50.7% in perceived enjoyment. Perceived friendliness showed the largest feature–need association with relatedness, whereas response timeliness was associated with autonomy and competence but not with relatedness. Autonomy and competence showed statistically significant direct and indirect associations with continuance intention, whereas relatedness showed a statistically significant indirect association involving perceived enjoyment but no significant direct association with continuance intention. Perceived enjoyment was positively associated with continuance intention and was involved in statistically significant indirect associations for all three psychological needs. These findings reveal differentiated feature–need and need–intention association patterns in physical AI educational hardware and suggest that design attention may usefully extend beyond responsiveness to relational and enjoyable interaction. Because the data are cross-sectional, these association patterns should not be interpreted as evidence of temporal or causal relationships.

## Introduction

As artificial intelligence technologies such as natural language processing and adaptive learning have matured, educational products are undergoing a transformation in form [1,2]. Unlike the static presentation of knowledge in conventional electronic learning aids, a new generation of intelligent educational products is evolving from purely functional tools toward intelligent learning companions with anthropomorphic interaction features, enabled by capabilities such as real-time dialogue, personalized content delivery, and affective feedback [3,4]. This shift has altered the nature of interaction between users and educational technology and has brought intelligent educational products into everyday home learning on a large scale [5–7].

However, upgrades in product form do not automatically translate into sustained user engagement. In practice, intelligent educational products commonly face a problem: users tend to show strong interest shortly after purchase, but their enthusiasm declines quickly after a period of interaction, and the product is subsequently left unused. Longitudinal studies of long-term interaction with children’s and educational intelligent products indicate that engagement decline driven by the novelty effect is common for this product category, with perceived anthropomorphism peaking at first contact and then declining systematically [8,9]. In the home setting, the long-term usage trajectory of a device is further shaped jointly by patterns of family involvement and individual differences among children [5]. Within the information systems research tradition, continuance intention is regarded as a central indicator of the realization of product value, exceeding initial adoption in importance [10], and it has been widely examined across e-learning and AI service contexts [11,12]. Understanding and improving users’ continuance intention toward intelligent educational products has become a shared concern for educational technology research and industry practice.

The AI Learning Device (AILD) is a representative case of the problem outlined above. An AILD is a category of physical educational hardware that integrates large language models, an adaptive learning engine, and multimodal interaction capabilities, oriented primarily toward the self-directed home learning of K-12 children. Compared with software-only AI educational applications, AILDs have three notable characteristics. First, as a tangible device with a relatively high purchase cost, an AILD makes the consequence of discontinued use particularly salient: once the device is left idle, the money and effort already invested cannot be recovered. This makes sustained use, rather than initial adoption, the critical outcome for this product category. Second, in the home setting the purchase is decided by parents while children are the primary users, and use is not externally mandated. Third, AILDs combine interaction-related features such as personalized recommendation, immediate feedback, conversational interaction, and friendly expression; some of these features also convey anthropomorphic and relational cues [4,13]. Taken together, the high investment, the non-mandatory home-learning setting, and the relational interaction cues make AILDs a distinct and underexamined context for investigating continuance intention.

Research on user behavior toward intelligent educational products has developed along two main lines. The first line draws on the Technology Acceptance Model (TAM) and the Expectation-Confirmation Model (ECM) of information systems continuance, focusing on cognitive and instrumental variables such as perceived usefulness and perceived ease of use [1,10]. The second line has more recently incorporated Self-Determination Theory (SDT), explaining users’ acceptance and continued use of AI applications through the three basic psychological needs of autonomy, competence, and relatedness, with empirical support in contexts such as chatbots, generative AI, and mobile learning [14–18]. These two lines have advanced understanding of user behavior toward AI applications, yet several questions remain open in the case of AILDs. Existing SDT research has mostly examined software-only AI, and systematic tests on physical educational hardware are relatively limited. Prior continuance-intention studies have often modeled the three psychological needs as parallel predictors, with limited examination of whether their direct associations with continuance intention and indirect associations involving perceived enjoyment differ [14]. In anthropomorphic AI contexts, perceived enjoyment has been found in several studies to be closely associated with continuance intention [19–21], but indirect association patterns involving psychological need satisfaction, perceived enjoyment, and continuance intention have not been systematically examined in the AILD context.

The design problem addressed here is therefore not the physical form of the device per se, but sustained engagement in a non-mandatory home-learning setting where functional assistance and relational cues are combined. Although the empirical setting of this study is China, the underlying design problem is not country-specific. Dedicated AI tutors, learning tablets, and socially interactive educational devices increasingly combine functional assistance with relational cues across home-learning contexts worldwide. Examining whether responsiveness, friendliness, and psychological need satisfaction show differentiated associations with continuance intention can therefore inform research on sustained engagement with AI-enabled educational products beyond the specific devices and market examined here.

The discussion sections of prior studies have repeatedly suggested that future work examine the psychological processes associated with anthropomorphic AI features and users’ long-term behavior [13,15], and longitudinal studies of children’s and educational intelligent products have likewise called for closer analysis of the specific psychological processes evoked by different product features [5,8]. In the AILD context, these suggestions have not yet been systematically addressed. When a physical educational device is used in the home over an extended period, it remains unclear which product features are associated with different psychological needs, whether the three needs show differentiated associations with continuance intention, and how perceived enjoyment is involved in these associations; these questions still lack integrated empirical examination. Answering them bears on extending SDT to the context of socially interactive AI-enabled educational products and on the long-term value realization of AILDs in home settings. Building on these considerations, this study integrates the Stimulus-Organism-Response (S-O-R) framework [22] with Self-Determination Theory [23,24] and positions perceived enjoyment as an affective construct for examining indirect associations [19,21], posing the following three research questions: (RQ1) Which interaction-related features of an AILD are treated as the relevant external stimuli, and what makes them distinctive in the LLM-enabled AILD context? (RQ2) How are these product features associated with users’ autonomy, competence, and relatedness needs, respectively? (RQ3) What differentiated association patterns emerge across the three psychological needs and continuance intention, and what indirect associations are observed through perceived enjoyment?

The directional hypotheses below specify broadly expected positive associations. Because prior evidence did not provide a sufficiently strong basis for specifying comparative differences across all feature–need and need–intention paths a priori, differences in coefficient magnitude and unsupported paths are interpreted as exploratory empirical patterns rather than as confirmatory tests of theoretically pre-specified differentiation.

For research, this study examines the underexplored context of dedicated physical AI learning devices and evaluates how interaction-related product features are associated with continuance intention through basic psychological needs and perceived enjoyment. Beyond testing the broadly specified directional hypotheses, the analysis also identifies exploratory differences in the estimated association patterns. Autonomy and competence showed both direct and indirect associations involving perceived enjoyment, whereas relatedness showed an indirect-only association through perceived enjoyment. At the feature level, perceived friendliness showed the strongest association with relatedness, while response timeliness was associated mainly with autonomy and competence. These empirically identified patterns provide a more differentiated descriptive account of need-specific associations in sustained engagement with AI-enabled educational products. For practice, the results suggest that responsiveness and friendliness may serve different experiential functions and that attention to functional performance may usefully be complemented by relational and enjoyable interaction design [13,25].

## Theoretical background and hypotheses

### AI learning devices

An AI Learning Device (AILD) is a category of physical educational hardware that integrates large language models, an adaptive learning engine, and multimodal interaction capabilities, oriented toward the self-directed home learning of K-12 children. In the present study, the AILDs used by respondents fell into two main forms: intelligent learning tablets and dedicated large-model-based AI learning assistants.

The distinctiveness of AILDs for user behavior research lies in three aspects. First, as physical hardware, an AILD has physical presence; its purchase cost and the sunk cost of use are markedly higher than those of software-only applications, so leaving it idle wastes the resources invested. Second, AILD use occurs in the home, where parents make the purchase decision and children are the primary users; the process lacks the external constraints and supervision common in classrooms or workplaces, so continued use depends more on users’ own intrinsic motivation [5]. Third, AILDs combine interaction-related features such as personalized recommendation, immediate feedback, conversational interaction, and friendly expression. Some of these features, particularly conversational style and friendly expression, also convey anthropomorphic and relational cues, so users may attend not only to functional performance but also form social perceptions and affective responses [3,4].

A clarification is warranted on why these four features, rather than generic system attributes, are treated as the relevant stimuli in the AILD context. Personalization, interactivity, responsiveness, and friendliness are not unique to AI products in the abstract; what distinguishes them here is the way large language models and an adaptive learning engine reconstitute each of them. Personalization in an AILD is driven by an adaptive engine that generates and adjusts content from the learner’s accumulated records, rather than retrieving items from a fixed rule-based bank as in conventional electronic learning aids. Interactivity takes the form of genuine multi-turn dialogue in which the user can follow up and the system re-explains in a different way, going beyond the single-pass delivery or fixed question-answer trees of pre-LLM devices. Response timeliness becomes a salient experiential dimension of generative, conversational feedback rather than merely a matter of loading speed. Friendliness, conveyed through natural language with discernible tone and warmth, becomes a designable interaction style that was largely unavailable on earlier educational hardware [4,26]. It is in this LLM-enabled form that the four features constitute the stimulus set examined in this study.

Prior studies indicate that anthropomorphic perceptions of AI systems may decline as the novelty effect wears off [8,9], whereas the formation of affective bonds can help sustain users’ long-term engagement to some extent [6]. However, existing research on user behavior toward intelligent educational products has mostly examined software-only applications such as chatbots and generative AI [14,16,17], and theoretical and empirical work on AILDs as physical educational hardware remains limited. This study therefore takes AILDs as the object of investigation and examines the associations among their product features, psychological need satisfaction, affective experience, and users’ continuance intention.

### Theoretical foundation

The Stimulus-Organism-Response (S-O-R) framework was originally proposed in environmental psychology by Mehrabian and Russell [22], positing that external environmental stimuli (Stimulus) influence an individual’s internal psychological state (Organism), which in turn elicits a behavioral response (Response). Owing to its three-stage structure, the framework has been adopted in information systems and user behavior research to explain how the features of technology products influence continued use through users’ cognitive and affective states [19,27–29]. In this study, the S-O-R framework provides the overall analytical structure: the product features of an AILD constitute the stimulus layer, users’ psychological need satisfaction and affective experience constitute the organism layer, and continuance intention constitutes the response layer.

Self-Determination Theory (SDT), proposed by Deci and Ryan [24], is a widely used theory for explaining human intrinsic motivation. SDT holds that the satisfaction of three basic psychological needs, autonomy, competence, and relatedness, is a key condition for activating intrinsic motivation: autonomy refers to an individual’s sense of volitional choice and control over behavior; competence refers to the sense of efficacy and capability experienced in interacting with the environment; and relatedness refers to the sense of connection with and care from others (or a system) [23]. In the technology use domain, the satisfaction of the three needs has been found to positively affect continued use of e-learning systems [30,31], engagement with AI chatbots [14,17], and acceptance of generative AI [15,16]. Peters et al. further noted that technology can both support and undermine the satisfaction of users’ basic psychological needs, and that specific features of a technology’s design determine the direction and degree of need satisfaction [32]. In this study, SDT is embedded in the organism layer of the S-O-R framework to examine how AILD product features are associated with users’ three basic psychological needs.

In sum, this study uses S-O-R as the overall framework and the three basic psychological needs of SDT as the core psychological constructs, specifying a theoretically ordered association model comprising product features, psychological need satisfaction, perceived enjoyment, and continuance intention. The stimulus layer comprises four product-feature variables: perceived personalization (PP), perceived interactivity (PI), response timeliness (RESP), and perceived friendliness (PF). The front end of the organism layer comprises the three psychological needs, autonomy (AUT), competence (COM), and relatedness (REL), and the back end comprises perceived enjoyment (ENJ) as the affective construct through which indirect associations are examined; the response layer is continuance intention (CI). In addition, because the three needs may be associated with usage intention both directly and indirectly through perceived enjoyment, the model retains direct paths from the three needs to CI. The following sections derive each construct and its associated hypotheses in turn.

### Constructs and hypotheses

#### Perceived personalization.

Perceived personalization (PP) refers to users’ perception of the degree to which a system tailors content and services to their individual characteristics, learning progress, and preferences [33–35]. In the AILD context, personalization is reflected mainly in functions such as automatically adjusting difficulty to the user’s level, recommending content suited to individual needs, and offering adjustment suggestions based on prior learning records.

Personalization may be positively associated with autonomy because a system that recognizes and respects individual differences may correspond to a stronger sense of volitional choice and control [17]. It may also be positively associated with competence because adaptive difficulty adjustment can correspond to an “I can do this” sense of efficacy [30]. Finally, personalization may be positively associated with relatedness when a system appears to “remember me” and “understand my needs,” experiences that may correspond to feeling attended to and understood [36,37]. We therefore propose:

H1a: Perceived personalization is positively associated with users’ autonomy.
H1b: Perceived personalization is positively associated with users’ competence.
H1c: Perceived personalization is positively associated with users’ relatedness.

#### Perceived interactivity.

Perceived interactivity (PI) refers to users’ perception of the degree of real-time, two-way, mutually responsive communication between the system and themselves [38,39]. In the AILD context, interactivity is reflected in question-and-answer exchanges between user and system, the system rephrasing its explanation when the user moves to a new question, and the user feeling engaged in a dialogue rather than passively receiving information.

Interactivity may be positively associated with autonomy because users can perceive that their input corresponds to changes in the system’s feedback and experience a sense that “I am directing this process” [40]. Interactivity may also be positively associated with competence because repeated exchanges provide opportunities for feedback and correction that can correspond to greater confidence in learning ability [30]. In addition, the dialogic, two-way nature of interaction may be positively associated with relatedness through the sense of social presence that “someone is communicating with me” [19]. We therefore propose:

H2a: Perceived interactivity is positively associated with users’ autonomy.
H2b: Perceived interactivity is positively associated with users’ competence.
H2c: Perceived interactivity is positively associated with users’ relatedness.

#### Response timeliness.

Response timeliness (RESP) refers to users’ perception of the speed and immediacy with which the system provides feedback after receiving input [41]. In the AILD context, timeliness is reflected in fast system replies, prompts or corrections delivered as soon as they are needed, and a pace that does not slow down the user’s problem-solving and learning.

Response timeliness may be positively associated with autonomy because immediate feedback can correspond to users’ perception of being in control of the interaction process [42]. It may also be positively associated with competence because rapid correction can correspond to perceptions of learning efficiency and self-efficacy [36]. Response speed, however, is primarily an attribute of functional efficiency that reflects system capability rather than warmth. Prior work suggests that users attribute timeliness more strongly to system capability than to emotional care [4]. Even so, immediate availability may also be associated with relatedness under conditions in which users experience the system as continuously responsive or socially present. We therefore retain a positive association hypothesis between response timeliness and relatedness:

H3a: Response timeliness is positively associated with users’ autonomy.
H3b: Response timeliness is positively associated with users’ competence.
H3c: Response timeliness is positively associated with users’ relatedness.

#### Perceived friendliness.

Perceived friendliness (PF) refers to users’ perception of the approachability, goodwill, and emotional warmth conveyed by an AILD during interaction. The construct is grounded in the warmth-competence framework of social cognition [43] and corresponds, in human-computer interaction, to the warmth dimension of a system’s interaction style [44]. Anthropomorphic cues such as language style and conversational manner have been shown to shape users’ social perceptions and affective responses toward AI systems [45–47]. In the AILD context, friendliness is reflected in the system feeling approachable, being friendly toward the user, and offering patient guidance when the user makes mistakes [26].

Bai et al. found that, in the multimodal interaction of anthropomorphic AI, the influence of perceived warmth on users’ positive evaluations strengthened with continued use, whereas the influence of competence-related perceptions weakened over time, so that warmth became relatively more important in longer-term interaction [13]. McKee et al. further showed that users’ social-cognitive evaluations of AI systems follow the warmth-competence framework, with perceived warmth closely associated with users’ trust and willingness to engage [4]. Conversely, AI services that lack warmth may produce a sense of distance and lower continuance intention [25].

Perceived friendliness may be positively associated with autonomy because a respectful and encouraging interaction style can correspond to a stronger perception that learning is voluntary [32]. Friendliness may also be positively associated with competence when encouragement and patient guidance correspond to greater confidence in facing learning challenges. Most directly, friendliness may be positively associated with relatedness because warmth and supportive expression correspond closely to the relational experience of being “treated kindly” and “accompanied” [13]. We therefore propose:

H4a: Perceived friendliness is positively associated with users’ autonomy.
H4b: Perceived friendliness is positively associated with users’ competence.
H4c: Perceived friendliness is positively associated with users’ relatedness.

#### Autonomy.

Autonomy (AUT) refers to the sense of volitional choice and control an individual experiences during behavior, and is one of the three basic psychological needs in SDT [23,24]. In the AILD context, autonomy is reflected in users being able to learn at their own pace, choose learning content and methods on their own, and feel that their learning is driven mainly by their own will rather than by compulsion.

In the technology use domain, autonomy need satisfaction has been positively associated with continuance-related outcomes across various settings. Roca and Gagné reported that autonomy need satisfaction was associated with e-learning continuance intention [30]. In an AI chatbot setting, Hidayat-ur-Rehman likewise reported a positive association between perceived autonomy and students’ learning engagement [17]. In the AILD context, a stronger sense of volitional choice may therefore be positively associated with users’ intention to continue using the device.

At the same time, SDT links autonomy need satisfaction with positive affective experience [23]. In technology-use contexts, a sense of control and freedom to proceed at one’s own pace has been associated with more pleasant and enjoyable experiences [30]. We therefore propose:

H5a: Autonomy is positively associated with perceived enjoyment.
H5b: Autonomy is positively associated with continuance intention.

#### Competence.

Competence (COM) refers to the sense of efficacy and capability an individual experiences in interacting with the environment, and is one of the three basic psychological needs in SDT [23,24]. In the AILD context, competence is reflected in users feeling that the device helps them grasp knowledge points more easily, makes learning more manageable, and gives them greater confidence in solving similar problems.

In educational technology settings, competence need satisfaction has been associated with persistence and positive technology-use outcomes. Xia et al. reported an indirect association involving competence need satisfaction in chatbot-assisted learning [14], while Bergdahl et al. found that competence need satisfaction was positively associated with users’ attitudes toward AI across six countries [15]. In the AILD context, a stronger “I can do this” sense of efficacy may therefore be positively associated with continuance intention.

Competence need satisfaction is also theoretically associated with positive achievement emotions [23]. Perceptions of learning efficacy and mastery may therefore be positively associated with pleasant and enjoyable experiences during technology use [30]. We therefore propose:

H6a: Competence is positively associated with perceived enjoyment.
H6b: Competence is positively associated with continuance intention.

#### Relatedness.

Relatedness (REL) refers to the experience of connection with, care from, and being understood by others (or a system), and is one of the three basic psychological needs in SDT [23,24]. In the AILD context, relatedness is reflected in users feeling that the system understands their confusion and learning needs, that they are not learning alone, and that someone is paying attention to their progress.

In the technology use domain, the association between relatedness and continued use appears to be more variable than those involving autonomy and competence. He and Li reported a positive association between relatedness and continuance intention in a mobile-learning setting [18], whereas Xia et al. reported a non-significant indirect association involving relatedness in an AI chatbot setting [14]. These findings suggest that relatedness need not show a consistent direct association with continuance intention across technology-use contexts. In the AILD context, the relational experience that a system “understands me” and “keeps me company” may nevertheless be positively associated with continuance intention.

At the same time, relatedness need satisfaction centers on the relational experience of being connected and cared for, which is more affective in emphasis than the efficacy-oriented experiences associated with autonomy and competence [23]. In socially interactive AI contexts, feeling that a system is “like a learning companion that understands me” may therefore be positively associated with affective experiences such as enjoyment and closeness [13]. We therefore propose:

H7a: Relatedness is positively associated with perceived enjoyment.
H7b: Relatedness is positively associated with continuance intention.

#### Perceived enjoyment.

Perceived enjoyment (ENJ) refers to the pleasant, interesting, and enjoyable affective state users experience while using a technology product [48]. The construct occupies a distinct explanatory role in information systems research on user behavior. Van der Heijden noted, in a model of hedonic information systems acceptance, that when the use of a technology product is itself hedonic in nature, perceived enjoyment rather than perceived usefulness becomes the central predictor of continuance intention [21]. Venkatesh et al. likewise incorporated hedonic motivation as an independent behavioral predictor in the UTAUT2 model [49]. More recently, perceived enjoyment has been positively associated with continuance intention in settings such as digital museums [19] and intelligent learning platforms [20].

From the perspective of SDT, satisfaction of the three basic psychological needs is theoretically associated with intrinsic motivation and with experiential states such as enjoyment and interest [23]. Roca and Gagné reported an indirect association between need satisfaction and continuance intention through perceived playfulness in an e-learning context [30]. Accordingly, the present model positions perceived enjoyment as an affective construct through which statistical indirect associations between psychological need satisfaction and continuance intention can be examined. This ordering is theoretically specified but does not, in the present cross-sectional design, imply temporal or causal mediation.

In the AILD context, users who experience greater pleasure and enjoyment during learning may also report stronger intentions to continue using the device. We therefore propose:

H8a: Perceived enjoyment is positively associated with continuance intention

#### Continuance intention.

Continuance intention (CI) refers to a user’s subjective intention to continue using an information system or technology product after initial adoption [10]. In the Expectation-Confirmation Model of information systems continuance, Bhattacherjee argued that continued use rather than initial adoption is the key indicator of information system success, because a product’s commercial and social value depends on long-term user retention rather than a one-time download or purchase [10]. This argument is especially salient in the AILD context: as physical educational hardware with a relatively high purchase cost, an AILD’s value realization depends heavily on whether users move from “using it for a few days after bringing it home” to “integrating it into daily learning over the long term”; once use is interrupted and the device is left idle, the economic and time costs already invested cannot be recovered [5]. In the S-O-R model of this study, continuance intention sits at the response layer as the final outcome construct, with hypothesized direct associations involving the three psychological needs (H5b, H6b, H7b) and indirect associations involving perceived enjoyment.

**Fig 1:**
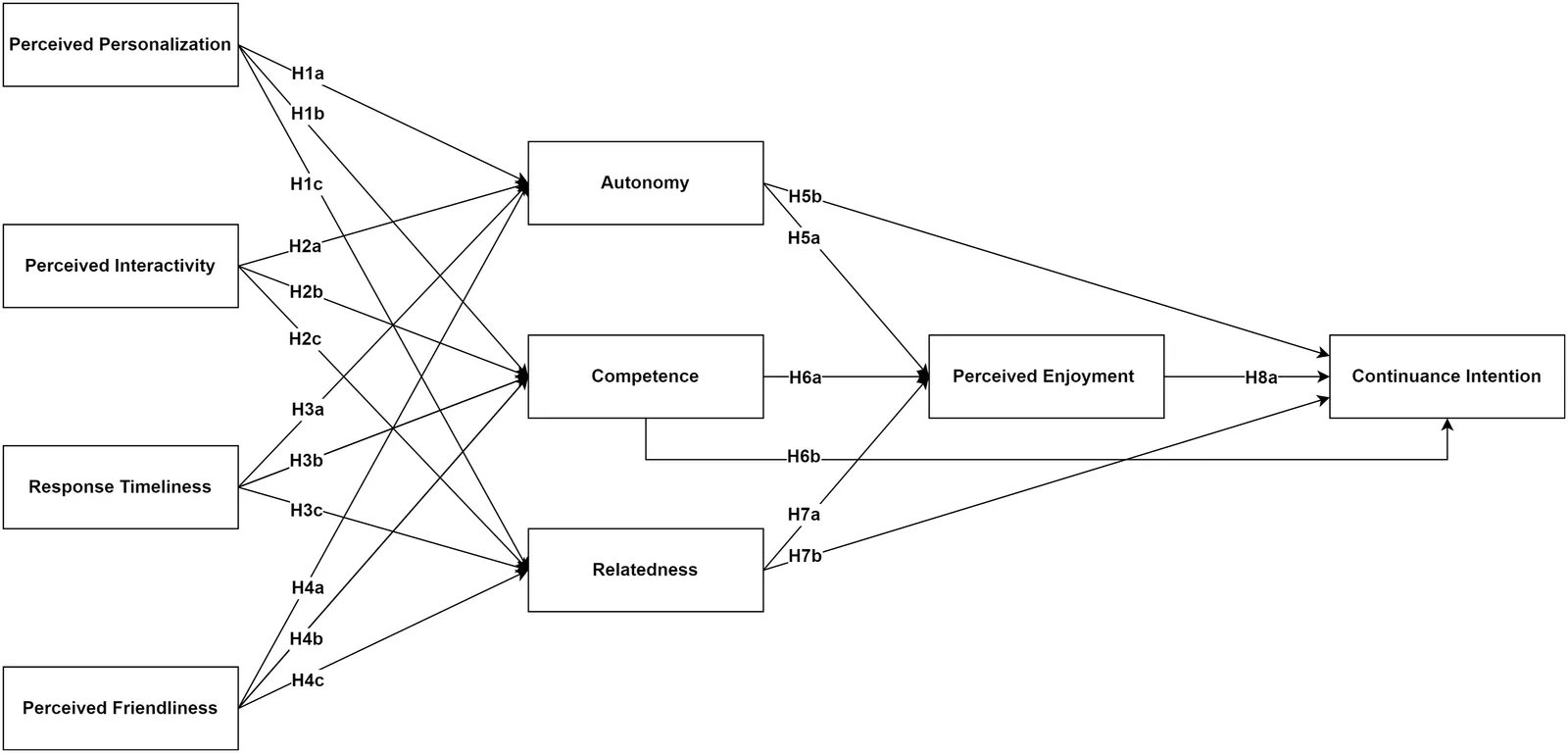
The proposed research model.

The proposed research model is shown in Fig 1.

## Method

Because the data are cross-sectional, the hypothesized paths are interpreted throughout as associations consistent with the proposed theoretical ordering rather than as established causal effects.

### Measurement instrument

All latent variables in this study were measured using validated scales from prior literature, adapted to the specific use context of AI Learning Devices. Items for all constructs used a five-point Likert scale ranging from 1 (strongly disagree) to 5 (strongly agree).

Perceived personalization (PP) was measured with three items adapted from the personalization scale of Tam and Ho [33], covering the degree to which the system tailors learning content, difficulty, and suggestions to the individual. Perceived interactivity (PI) was measured with three items adapted from the interactivity scales of Liu [38] and Song and Zinkhan [39], capturing users’ experience of real-time, two-way communication with the AILD. Response timeliness (RESP) was measured with three items (RT1 to RT3) drawn from the responsiveness dimension of the SERVQUAL scale [50] and the robotic service quality scale of Prentice and Nguyen [41], reflecting the speed and immediacy of the system’s feedback to user input. Perceived friendliness (PF) was measured with three items adapted from the likeability dimension of the Godspeed scale [44] and the warmth dimension of social cognition [43], capturing users’ perception of the friendliness of the AILD’s expression and interaction style.

The three psychological-need constructs, autonomy (AUT), competence (COM), and relatedness (REL), were based on the Basic Psychological Needs Scale (BPNS) [51] and its adaptation to online learning by Chen and Jang [52], with three items each, measuring respectively the room for volitional choice, the sense of operational efficacy, and the sense of affective connection that users experienced during use. Perceived enjoyment (ENJ) was measured with three items adapted from Davis et al. [48] and Van der Heijden [21], reflecting the degree of pleasure and enjoyment users experienced during use. Continuance intention (CI) was measured with three items adapted from the information systems continuance scale of Bhattacherjee [10], capturing users’ subjective intention to continue using the product in the future. These nine constructs, comprising 27 items in total, constituted the formal measurement model of this study. The full list of measurement items, with their sources, is given in Table 1.

**Table 1:** Measurement constructs, items, and sources.

Construct	Item	Description	Source
Perceived Personalization	PP1	It adjusts the difficulty to my level.	[33]
	PP2	The content it recommends fits my needs.	
	PP3	It draws on my past learning to adjust its suggestions.	
Perceived Interactivity	PI1	I can have a back-and-forth exchange with it.	[38,39]
	PI2	When I rephrase a question, it explains in a different way.	
	PI3	It feels like a conversation rather than one-way delivery.	
Response Timeliness	RT1	When I ask, it replies quickly.	[41,50]
	RT2	When I need it, it gives prompts or corrections right away.	
	RT3	It does not slow down my problem-solving or learning pace.	
Perceived Friendliness	PF1	It feels approachable.	[43,44]
	PF2	It encourages me to keep learning.	
	PF3	It speaks respectfully and does not make me uncomfortable.	
Autonomy	AUT1	When studying with it, I can go at my own pace.	[51,52]
	AUT2	When studying with it, I can choose what and how to learn.	
	AUT3	Studying with it is mainly my own choice, not something imposed.	
Competence	COM1	It helps me grasp knowledge points more easily.	[51,52]
	COM2	It makes my learning feel more manageable.	
	COM3	After using it, I feel more confident with similar problems.	
Relatedness	REL1	I feel it understands my confusion and learning needs.	[51,52]
	REL2	When studying with it, it does not feel like learning alone.	
	REL3	When studying with it, I feel someone is paying attention to my progress.	
Perceived Enjoyment	ENJ1	Studying with it makes me feel happy and relaxed.	[21,48]
	ENJ2	Studying with it is interesting.	
	ENJ3	I enjoy using it to study.	
Continuance Intention	CI1	I intend to keep using it to study in the future.	[10]
	CI2	When I have a learning need, I will use it first.	
	CI3	I will use it more often in the future.	
Privacy Concern (not analyzed)	PRI1	I worry it collects too much of my information.	
	PRI2	I worry my information is used for things I am not aware of.	
	PRI3	I worry my information could be leaked.	

*Note.* All items were rated on a five-point Likert scale (1 = strongly disagree to 5 = strongly agree). The three privacy-concern items were collected for a separate study and were not analyzed in this paper.

The questionnaire also included three items on privacy concern that were collected for a separate study and were not analyzed here.

The original English items were translated into Chinese and independently back-translated by two bilingual researchers; discrepancies were reconciled through discussion to ensure semantic equivalence with the source scales. Before formal administration, three researchers in human-computer interaction and two parents with experience using AILDs assessed the content validity of the items, and the wording of several items was revised accordingly so that it matched the everyday usage context of the target group. Because the respondents were first-year senior high school students, the instructions were framed in an approachable tone as “a letter to a young researcher,” and each construct used item wording close to students’ everyday expression (for example, “When I study with it, I can go at my own pace” and “Its explanations are clear and easy to follow”) to reduce comprehension load and improve response quality.

### Data collection

#### Ethical review.

This study was conducted in accordance with the principles of the Declaration of Helsinki and received ethical approval from the Institutional Review Board of the School of Design Arts, Xiamen University of Technology (Approval Number: XMUT-SDA-IRB-2025-12/009). All procedures were carried out in accordance with the relevant guidelines and regulations governing research with minors in educational settings in China. Because the participants were minors (first-year senior high school students aged 15–16), written informed consent was obtained from the participants’ parents or legal guardians, and written assent was obtained from the participants themselves, prior to questionnaire administration. School administrators and the parents of all participating students also provided permission for the research to be conducted on school premises during regular class time. The consent and assent materials stated the purpose of the study, the principle of voluntary participation, the procedures for anonymizing data, and the right to withdraw, ensuring that participants decided whether to take part after being fully informed.

#### Sampling.

The study targeted first-year senior high school students at a public, science-and-technology-oriented high school in Xiamen, Fujian Province, China. This group was selected for three reasons. First, first-year senior high school students are at a stage where academic pressure rises markedly, and AILDs as exam-preparation aids have a relatively high household penetration rate in this group, which provided access to a sufficient number of students with actual AILD experience. Second, this group has the cognitive capacity to operate an AILD independently and to form stable usage experience and judgment, and can therefore provide valid evaluations of constructs such as perceived personalization, interactivity, and friendliness. Third, the narrow age range of these students (about 15–16 years) yields relatively uniform usage contexts, which helps control heterogeneity arising from differences in age and grade level.

#### Data collection procedure.

Data were collected in May 2026 using paper questionnaires administered on site in group sessions. After coordinating with the participating school, the research team organized students to respond together in a lecture hall or multifunction auditorium at the school. Teachers present at each session maintained order and explained procedural questions about completion (such as the meaning of the scale points and how to turn pages); they did not interpret or guide the content of any item, so as to avoid social desirability effects.

On-site group administration was chosen for three reasons. First, completing paper questionnaires at a uniform time and place keeps the response environment consistent and reduces the environmental noise common in online distribution. Second, the presence of teachers ensures that respondents were the intended first-year senior high school students themselves, safeguarding sample validity at the source. Third, group administration helps secure a high response rate and completion quality and lowers the risk of dropout or proxy completion.

Before completing the questionnaire, respondents were informed that an AILD refers specifically to a dedicated physical device designed primarily for AI-supported learning (such as an intelligent learning tablet or a large-model-based AI learning assistant), and that general-purpose large language model applications accessed through smartphones, personal computers, or web browsers (e.g., ChatGPT, DeepSeek, Doubao) were not included. The first item was then a screening question, “Have you used, or are you currently using, a dedicated AI learning device for studying?” Respondents answered with reference to the specific AILD they had personally used. Only questionnaires from respondents who answered “yes” were included; those whose experience was limited to general-purpose LLM software were excluded. After collection, the research team checked each questionnaire and excluded those that failed the screening question, had extensive missing data (three or more missing items), or showed patterned responding (such as selecting the same option throughout or a clear zigzag pattern).

A total of 780 questionnaires were distributed and 778 were returned (a return rate of 99.7%). After the screening procedure above, 726 valid questionnaires were obtained, for an effective response rate of 93.1%.

The demographic characteristics of the sample are summarized in Table 2. There were 372 male students (51.2%) and 354 female students (48.8%), a relatively balanced gender distribution. In terms of usage duration, 48.1% had used the product for more than one year, 16.8% for 6–12 months, 14.9% for 3–6 months, 10.9% for 1–3 months, and 9.4% for less than one month. In terms of usage frequency, 12.0% used it daily, 44.4% several times a week, 14.5% several times a month, 28.0% occasionally, and 1.2% rarely. In terms of device type, 72.6% used a learning device or learning tablet and 27.4% used a dedicated, large-model-based AI learning assistant.

**Table 2:** Demographic information of participants.

Demographics	Item	Subjects (N = 726)	
		Frequency	Percentage
Gender	Male	372	51.2%
	Female	354	48.8%
Usage duration	Less than 1 month	68	9.4%
	1–3 months	79	10.9%
	3–6 months	108	14.9%
	6–12 months	122	16.8%
	More than 1 year	349	48.1%
Usage frequency	Daily	87	12.0%
	Several times a week	322	44.4%
	Several times a month	105	14.5%
	Occasionally	203	28.0%
	Rarely	9	1.2%
Device type	Learning device / learning tablet	527	72.6%
	Dedicated AI learning assistant device (large-model-based)	199	27.4%

It should be noted that respondents evaluated the specific AILD they had personally used rather than a single standardized device presented by the researchers. As the demographic profile shows, these devices spanned two main forms, namely intelligent learning tablets and dedicated large-model-based AI learning assistants, as well as various brands and models, so the stimulus layer of the model was not experimentally controlled across respondents. This was a deliberate design choice rather than an oversight. Standardizing the stimulus to one device would have increased internal control at the cost of ecological validity, whereas the aim of this study was to examine how the interaction-related features shared by AILDs *as a product category* are associated with psychological need satisfaction in authentic home-learning settings, rather than to estimate the effect of any single product [5]. The constructs were therefore measured at a level of abstraction intended to apply across AILD forms rather than to capture device-specific implementations.

## Results

This study used partial least squares structural equation modeling (PLS-SEM), analyzed with SmartPLS 4.0. PLS-SEM was chosen over covariance-based SEM (CB-SEM) for two reasons. First, Cramér-von Mises tests indicated that all items deviated significantly from normality (*p* < .001), and PLS-SEM imposes weaker assumptions on the data distribution. Second, the model contains nine latent variables and multiple direct and indirect paths, and the analysis focuses on simultaneously estimating these associations and explaining variance in the endogenous constructs, for which PLS-SEM is well suited [53,54].

### Common method bias

Because both the independent and dependent variables came from self-report questionnaires completed by the same respondents, there was a potential risk of common method bias (CMB). To mitigate this risk, several procedural measures were adopted. The questionnaire was completed anonymously, the instructions stated that there were no right or wrong answers in order to reduce social desirability effects, and the item order was arranged appropriately. As a supplementary diagnostic, Harman’s single-factor test was conducted by entering all 27 items into an unrotated principal component analysis. The first factor accounted for 43.70% of the total variance, below the commonly used 50% threshold. Because Harman’s test is relatively insensitive, CMB was also examined using a full-collinearity assessment [55]. All full-collinearity VIF values were below 3.3. These diagnostics did not indicate an obvious severe common-method problem; however, neither test can establish the absence of CMB. Accordingly, the possibility that shared method variance inflated some of the observed associations cannot be excluded and is considered further in the Discussion and Limitations.

### Measurement model

#### Reliability and convergent validity.

As shown in Table 3, Cronbach’s α for the constructs ranged from 0.766 to 0.881, composite reliability $\rho_{a}$ from 0.767 to 0.881, and $\rho_{c}$ from 0.865 to 0.926, all above the 0.70 threshold, indicating adequate internal consistency reliability. For convergent validity, outer loadings of all items ranged from 0.792 to 0.917, all above 0.70, and the average variance extracted (AVE) of each construct ranged from 0.681 to 0.808, all above the 0.50 criterion. Reliability and convergent validity were supported, with all criteria following the recommendations of Hair et al. [53].

**Table 3:** Measurement model results: reliability and validity.

Construct	Items	Factor Loading	Cronbach’s Alpha	$\rho_{a}$	$\rho_{c}$	AVE
Perceived Personalization	PP1	0.834				
	PP2	0.849	0.793	0.794	0.878	0.707
	PP3	0.838				
Perceived Interactivity	PI1	0.855				
	PI2	0.867	0.821	0.821	0.894	0.737
	PI3	0.852				
Response Timeliness	RT1	0.792				
	RT2	0.862	0.766	0.767	0.865	0.681
	RT3	0.821				
Perceived Friendliness	PF1	0.836				
	PF2	0.898	0.819	0.820	0.893	0.735
	PF3	0.837				
Autonomy	AUT1	0.839				
	AUT2	0.891	0.819	0.818	0.892	0.734
	AUT3	0.840				
Competence	COM1	0.880				
	COM2	0.895	0.845	0.846	0.907	0.764
	COM3	0.846				
Relatedness	REL1	0.826				
	REL2	0.903	0.833	0.833	0.900	0.751
	REL3	0.869				
Perceived Enjoyment	ENJ1	0.894				
	ENJ2	0.917	0.881	0.881	0.926	0.808
	ENJ3	0.885				
Continuance Intention	CI1	0.857				
	CI2	0.846	0.806	0.816	0.885	0.719
	CI3	0.841				

*Note.* All factor loadings, $\rho_{c}$, and AVE exceed the recommended thresholds (0.70, 0.70, 0.50). Response Timeliness items are coded RT1–RT3.

#### Discriminant validity.

Discriminant validity was assessed using two criteria. For the Fornell-Larcker criterion, the square root of each construct’s AVE exceeded its correlations with the other constructs (see Table 4). For the heterotrait-monotrait ratio (HTMT), values between all construct pairs ranged from 0.470 to 0.809, below the 0.85 threshold recommended by Henseler et al. [56] (see Table 5). The highest HTMT value, between relatedness and perceived enjoyment (0.759), had a bootstrap 95% confidence interval of [0.697, 0.815] that excluded 1 and fell below 0.85, supporting their discriminant validity despite their conceptual proximity. The two criteria jointly indicate adequate discriminant validity among the constructs.

**Table 4:** Correlation of the research variables.

Construct	1	2	3	4	5	6	7	8	9
1. Perceived Personalization	0.841								
2. Perceived Interactivity	0.578	0.858							
3. Response Timeliness	0.608	0.613	0.826						
4. Perceived Friendliness	0.549	0.614	0.641	0.857					
5. Autonomy	0.478	0.531	0.593	0.552	0.857				
6. Competence	0.530	0.531	0.590	0.523	0.603	0.874			
7. Relatedness	0.504	0.517	0.474	0.566	0.390	0.615	0.867		
8. Perceived Enjoyment	0.481	0.517	0.499	0.556	0.496	0.586	0.650	0.899	
9. Continuance Intention	0.425	0.454	0.511	0.516	0.547	0.585	0.484	0.592	0.848

*Note.* Diagonal elements are the square root of AVE.

**Table 5:** HTMT ratio.

Construct	1	2	3	4	5	6	7	8
1. Perceived Personalization								
2. Perceived Interactivity	0.717							
3. Response Timeliness	0.778	0.773						
4. Perceived Friendliness	0.682	0.749	0.809					
5. Autonomy	0.592	0.648	0.750	0.671				
6. Competence	0.643	0.637	0.732	0.628	0.724			
7. Relatedness	0.620	0.623	0.591	0.686	0.470	0.731		
8. Perceived Enjoyment	0.576	0.607	0.609	0.656	0.584	0.680	0.759	
9. Continuance Intention	0.527	0.551	0.647	0.628	0.664	0.707	0.590	0.692

*Note.* All HTMT values are below the 0.85 threshold

#### Collinearity.

Indicator-level VIF values ranged from 1.449 to 2.868, below the threshold of 5, indicating no notable collinearity among the indicators. In addition, the inner-model VIF values for the predictor constructs ranged from 1.573 to 2.210, also below the threshold of 5, indicating that structural collinearity was not a concern.

### Structural model

After confirming the quality of the measurement model, the significance of the structural path coefficients was tested using 5,000 bootstrap resamples.

#### Explanatory power.

**Fig 2:**
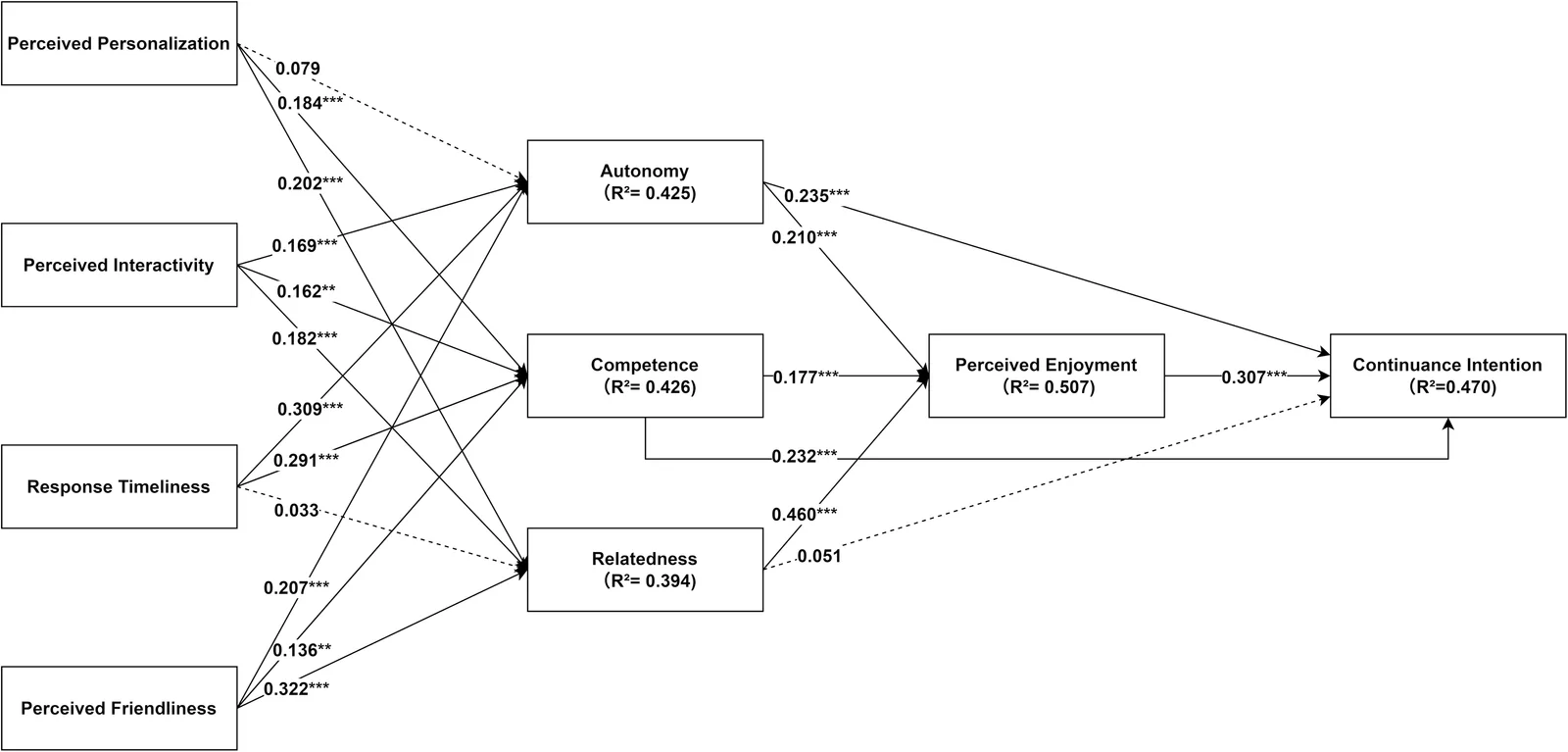
Results of the structural model. Note: Solid lines indicate statistically significant paths, whereas dashed lines indicate non-significant paths. Values on the paths are standardized path coefficients. ^***^ *p* < .001, ^**^ *p* < .01.

Fig 2 presents the results for all hypothesized structural paths, with non-significant paths shown as dashed lines. As shown in Table 6, the *R*^2^ was 0.507 for perceived enjoyment (ENJ) and 0.470 for continuance intention (CI); the *R*^2^ values for the three psychological needs were 0.426 for competence, 0.425 for autonomy, and 0.394 for relatedness. These values are at a moderate-to-high level, indicating that the selected product features and psychological needs account for a substantial share of the variance in each endogenous variable.

**Table 6:** Explanatory power (*R*^2^) of endogenous constructs.

Endogenous construct	*R* ^2^	$R_{adj}^{2}$
Autonomy	0.425	0.422
Competence	0.426	0.423
Relatedness	0.394	0.390
Perceived Enjoyment	0.507	0.505
Continuance Intention	0.470	0.467

#### Associations of product features with psychological needs.

Among the 12 associations between the four product features and the three psychological needs, perceived friendliness with relatedness showed the largest standardized coefficient (β=0.322, *p* < .001, *f*^2^ = 0.085), followed by response timeliness with autonomy (β=0.309, *p* < .001, *f*^2^ = 0.077) and competence (β=0.291, *p* < .001, *f*^2^ = 0.069). Two expected associations were not supported: perceived personalization with autonomy (β=0.079, *p* = .071, *f*^2^ = 0.006) and response timeliness with relatedness (β=0.033, *p* = .530, *f*^2^ = 0.001). The remaining statistically significant feature–need associations had small effect sizes, and the two unsupported associations are examined more closely in the Discussion because of their theoretical and design relevance.

#### Associations of psychological needs and perceived enjoyment with continuance intention.

The three psychological needs showed different association patterns with continuance intention. The direct association between relatedness (REL) and continuance intention (CI) was not statistically significant (β=0.051, *p* = .307), whereas relatedness showed the largest association with perceived enjoyment (ENJ) (β=0.460, *p* < .001). Relatedness also showed a statistically significant indirect association with continuance intention through perceived enjoyment. By contrast, autonomy (AUT) and competence (COM) showed statistically significant direct associations with continuance intention (AUT: β=0.235; COM: β=0.232; both *p* < .001) as well as statistically significant indirect associations through perceived enjoyment. Perceived enjoyment was positively associated with continuance intention (β=0.307, *p* < .001). These results describe statistical association patterns within the cross-sectional model and should not be interpreted as evidence of temporal or causal transmission.

#### Effect sizes.

Effect-size interpretation substantially qualifies the significance-based results. The association between relatedness and perceived enjoyment had the largest effect size (*f*^2^ = 0.266), representing a medium effect under conventional benchmarks. All remaining effects were small or negligible. The comparatively larger among these small effects were perceived enjoyment with continuance intention (*f*^2^ = 0.088), perceived friendliness with relatedness (*f*^2^ = 0.085), response timeliness with autonomy (*f*^2^ = 0.077) and competence (*f*^2^ = 0.069), and autonomy with continuance intention (*f*^2^ = 0.063). Several statistically significant paths carried only very small effects, including H4b (*f*^2^ = 0.016), H2b (*f*^2^ = 0.023), H2a (*f*^2^ = 0.025), H6a (*f*^2^ = 0.030), and H1b (*f*^2^ = 0.033). Given the sample size of 726, statistical significance alone should therefore not be interpreted as evidence of substantive importance. The interpretation below gives greater weight to effect sizes and to theoretically informative unsupported associations (Table 7).

**Table 7:** Summary of the structural model results.

H	Predictor	Outcome	β	*t*	*p*	*f* ^2^	Result
H1a	Perceived Personalization	Autonomy	0.079	1.803	.071	0.006	Not supported
H1b	Perceived Personalization	Competence	0.184	3.972	<.001	0.033	Supported
H1c	Perceived Personalization	Relatedness	0.202	4.270	<.001	0.037	Supported
H2a	Perceived Interactivity	Autonomy	0.169	3.527	<.001	0.025	Supported
H2b	Perceived Interactivity	Competence	0.162	3.229	.001	0.023	Supported
H2c	Perceived Interactivity	Relatedness	0.182	4.015	<.001	0.028	Supported
H3a	Response Timeliness	Autonomy	0.309	6.534	<.001	0.077	Supported
H3b	Response Timeliness	Competence	0.291	6.172	<.001	0.069	Supported
H3c	Response Timeliness	Relatedness	0.033	0.628	.530	0.001	Not supported
H4a	Perceived Friendliness	Autonomy	0.207	4.229	<.001	0.037	Supported
H4b	Perceived Friendliness	Competence	0.136	2.877	.004	0.016	Supported
H4c	Perceived Friendliness	Relatedness	0.322	6.691	<.001	0.085	Supported
H5a	Autonomy	Perceived Enjoyment	0.210	5.048	<.001	0.057	Supported
H5b	Autonomy	Continuance Intention	0.235	5.579	<.001	0.063	Supported
H6a	Competence	Perceived Enjoyment	0.177	3.588	<.001	0.030	Supported
H6b	Competence	Continuance Intention	0.232	4.778	<.001	0.046	Supported
H7a	Relatedness	Perceived Enjoyment	0.460	11.624	<.001	0.266	Supported
H7b	Relatedness	Continuance Intention	0.051	1.022	.307	0.002	Not supported
H8a	Perceived Enjoyment	Continuance Intention	0.307	5.943	<.001	0.088	Supported

*Note.* β = standardized path coefficient; significance based on 5,000 bootstrap resamples

#### Indirect-association analysis.

To examine the statistical indirect associations involving perceived enjoyment, specific indirect coefficients were estimated using bias-corrected bootstrapping with 5,000 resamples. An indirect association was considered statistically significant when its 95% confidence interval excluded zero [57]. All three estimated indirect associations through perceived enjoyment were statistically significant. The relatedness to perceived enjoyment to continuance intention sequence showed the largest indirect coefficient (β=0.141, 95% CI [0.090, 0.198]), followed by autonomy (β=0.065, 95% CI [0.035, 0.101]) and competence (β=0.054, 95% CI [0.026, 0.096]).

For descriptive purposes, the direct and indirect association patterns were compared following the classification framework of Zhao et al. [58]. Autonomy and competence each showed statistically significant direct and indirect associations with continuance intention, whereas relatedness showed a statistically significant indirect association together with a non-significant direct association (β=0.051, *p* = .307). These labels describe the pattern of coefficients in the estimated cross-sectional model only. They do not establish temporal ordering or causal mediation. The corresponding coefficients are reported in Table 8.

**Table 8:** Direct and indirect association patterns involving perceived enjoyment.

Path	Direct association β (*p*)	Indirect association β	*p*	95% CI	Association pattern
REL → ENJ → CI	0.051 (.307)	0.141	<.001	[0.090, 0.198]	Indirect-only pattern
AUT → ENJ → CI	0.235 (<.001)	0.065	<.001	[0.035, 0.101]	Direct + indirect pattern
COM → ENJ → CI	0.232 (<.001)	0.054	.002	[0.026, 0.096]	Direct + indirect pattern

*Note.* AUT = Autonomy; COM = Competence; REL = Relatedness; ENJ = Perceived Enjoyment; CI = Continuance Intention. Specific indirect associations were estimated with bias-corrected bootstrapping using 5,000 resamples; a 95% CI excluding zero indicates a statistically significant indirect association. The reported patterns describe coefficient relationships in the cross-sectional model and do not establish temporal or causal mediation.

#### Robustness check with control variables.

To assess whether the main associations were robust to usage experience and device type, we re-estimated the structural model with three control variables, usage duration, usage frequency, and device type, added as additional predictors of continuance intention. Usage frequency was recoded so that higher values represented more frequent use (1 = rarely, 2 = occasionally, 3 = several times a month, 4 = several times a week, and 5 = daily). Usage duration (β=0.011, *p* = .692) and device type (β=0.026, *p* = .657) were not significantly associated with continuance intention, whereas usage frequency showed a small positive association (β=0.117, *p* < .001). Adding these controls raised the explained variance in continuance intention only marginally (*R*^2^ from 0.470 to 0.483), and the core structural paths were virtually unchanged (e.g., AUT → CI: 0.228 vs. 0.235; COM → CI: 0.220 vs. 0.232; ENJ → CI: 0.301 vs. 0.307; REL → CI remained non-significant). These results indicate that including the three control variables did not materially alter the main association patterns. Full coefficients are reported in Table 9.

**Table 9:** Structural paths to continuance intention with control variables.

Predictor	Outcome	β	*t*	*p*	Result
Autonomy	Continuance Intention	0.228	5.431	<.001	Significant
Competence	Continuance Intention	0.220	4.633	<.001	Significant
Relatedness	Continuance Intention	0.059	1.216	.224	n.s.
Perceived Enjoyment	Continuance Intention	0.301	5.936	<.001	Significant
Usage duration	Continuance Intention	0.011	0.397	.692	n.s.
Usage frequency	Continuance Intention	0.117	4.305	<.001	Significant
Device type	Continuance Intention	0.026	0.444	.657	n.s.

*Note.* Control variables: usage duration (1 = < 1 month to 5 = > 1 year), usage frequency (1 = rarely, 2 = occasionally, 3 = several times a month, 4 = several times a week, 5 = daily), and device type (1 = AI learning assistant, 2 = learning device/tablet). Estimates based on 5,000 bootstrap resamples. Core path coefficients are essentially unchanged relative to the main model (Table 7), indicating robustness.

## Discussion

Building on the S-O-R framework, this study integrated Self-Determination Theory and perceived enjoyment to specify and test an association model involving AILD users’ perceptions of product features, psychological needs, perceived enjoyment, and continuance intention. The PLS-SEM analysis of 726 first-year senior high school students revealed heterogeneous association patterns across the estimated paths, with substantial variation in effect sizes and several theoretically informative non-significant associations. The following sections discuss the main model, its theoretical and practical implications, and the limitations of the study.

### Discussion of the main model

#### Differentiated associations between product features and psychological needs.

The hypotheses reflected broadly positive a priori expectations, because each product feature could plausibly relate to more than one psychological need. They were not designed as comparative hypotheses specifying that one feature–need association should be stronger than another. Accordingly, when the four features were estimated jointly, the differences in coefficient magnitude and the unsupported paths were interpreted as exploratory empirical patterns rather than as confirmation of a priori differentiated predictions.

Perceived friendliness (PF) showed the largest association coefficient among the stimulus–need paths, with relatedness (REL) (β=0.322), supporting H4c. This result is consistent with the expectation derived earlier, in the development of the perceived friendliness hypotheses, from the warmth-competence framework [4,43]: as a core indicator of the warmth dimension, friendliness was most strongly associated with the relational experience of being treated kindly and accompanied. The finding is in line with Bai et al., who reported that perceived warmth plays a distinct role in anthropomorphic AI interaction and becomes more important in long-term use [13].

The unsupported association between response timeliness and relatedness is particularly informative. Response timeliness was associated with autonomy (β=0.309, *f*^2^ = 0.077) and competence (β=0.291, *f*^2^ = 0.069), but showed essentially no association with relatedness (β=0.033, *p* = .530, *f*^2^ = 0.001). This pattern suggests that response speed should not be treated as a proxy for relational connection. Fast feedback may be experienced primarily as functional capability and efficiency, whereas feeling understood or accompanied may depend on qualitatively different interaction cues such as warmth, patience, and friendly expression.

The unsupported association between perceived personalization and autonomy is similarly noteworthy. Despite the common assumption that personalization should enhance users’ sense of control, perceived personalization showed only a very small and non-significant association with autonomy (β=0.079, *p* = .071, *f*^2^ = 0.006). One possible post hoc explanation is that personalization may sometimes be experienced as passive system adaptation, in which the system selects or adjusts learning content on the user’s behalf rather than expanding the user’s active choice. Research on the artificial autonomy of intelligent assistants similarly suggests that system-initiated decisions may partly substitute for users’ own decision space [59]. The present result therefore raises the possibility that adaptive personalization and perceived autonomy should not be treated as interchangeable design outcomes.

#### Exploratory differences in need–intention association patterns.

An exploratory finding of this study is that the three psychological needs showed different estimated association patterns with continuance intention. These differences were identified empirically rather than specified as comparative predictions during hypothesis development.

The direct association between relatedness and continuance intention was not statistically significant (H7b not supported, β=0.051, *p* = .307), whereas the association between relatedness and perceived enjoyment was the largest in the model (H7a supported, β=0.460, *p* < .001, *f*^2^ = 0.266). The bootstrap analysis also identified a statistically significant indirect association between relatedness and continuance intention through perceived enjoyment (β=0.141, 95% CI [0.090, 0.198]). Thus, within the estimated cross-sectional model, relatedness showed a significant indirect association together with a non-significant direct association. This coefficient pattern is consistent with the possibility that enjoyment is relevant to the association between relatedness and continuance intention, but it does not demonstrate temporal mediation or a causal process.

Unlike relatedness, autonomy and competence each showed statistically significant direct and indirect associations with continuance intention. In descriptive terms, this differs from the relatedness pattern, for which only the indirect association was statistically significant. Consistent with SDT [23], these results suggest that autonomy, competence, and relatedness may occupy different positions within the observed association structure. However, because all variables were measured concurrently, the direct and indirect coefficient patterns should not be interpreted as evidence that the needs temporally precede enjoyment or that enjoyment causally mediates their associations with continuance intention.

Taken together, the three psychological needs did not show homogeneous association patterns with continuance intention in the present sample: autonomy and competence showed statistically significant direct and indirect associations, whereas relatedness showed a statistically significant indirect association but no significant direct association. These differences should be regarded as descriptive patterns in the cross-sectional model rather than evidence of distinct causal pathways.

#### Perceived enjoyment in the indirect association structure.

Perceived enjoyment was positively associated with continuance intention (H8a supported, β=0.307, *p* < .001). In the estimated model, perceived enjoyment was also the construct through which all three psychological needs showed statistically significant indirect associations with continuance intention. This pattern is consistent with the proposed theoretical positioning of enjoyment as an affective correlate linking psychological need satisfaction and continuance intention, while the cross-sectional design precludes conclusions about temporal or causal mediation.

Among the three psychological needs associated with perceived enjoyment, relatedness showed the largest estimated coefficient (β=0.460), compared with autonomy (β=0.210) and competence (β=0.177). This ordering is worth examining because classic SDT accounts generally place greater emphasis on autonomy and competence in relation to intrinsic motivation and its experiential correlates, while relatedness is often conceptualized as a supportive condition [23]. A contextual interpretation is that AILDs are not purely instrumental tools but often incorporate relational cues intended to make the system feel like a study companion that “understands me” and “keeps me company.” In this context, the relational experience of being understood and accompanied may be more closely associated with enjoyment than experiences centered on control or competence. This reading is consistent with Bai et al., who found that perceived warmth becomes relatively more important than competence in longer-term anthropomorphic AI interaction [13]. An alternative, and not mutually exclusive, explanation concerns conceptual proximity and common method variance. Relatedness, which captures feeling accompanied and understood, and perceived enjoyment, which captures feeling happy and relaxed during use, are both affectively toned and were measured through the same self-report questionnaire. Their HTMT value of 0.759 was the highest in the matrix, although it remained below the 0.85 discriminant-validity threshold. Discriminant validity, however, does not eliminate the possibility of shared method variance. The relatively strong relatedness–enjoyment association may therefore reflect both substantive covariation and inflation arising from the shared measurement context. Its magnitude should consequently be interpreted cautiously and replicated using temporally separated, multi-source, or behavioral measures. Taken together, these results suggest that relational and affective experiences warrant attention in AILD research because relatedness showed a comparatively strong association with enjoyment, which was in turn positively associated with continuance intention. This pattern is broadly consistent with prior findings linking enjoyment with continued-use intention in intelligent learning platforms [19,20]. For design, the findings support considering relational qualities alongside functional performance, while causal effects on sustained use remain to be established experimentally or longitudinally.

### Implications for theory and practice

#### Theoretical implications.

The findings have three implications for theory.

First, the study broadens the empirical application of SDT to the underexamined context of dedicated physical AI learning devices. Prior research on SDT and AI user behavior has mostly examined software applications such as chatbots and generative AI [14,16,17]. By examining AILD users in a home-learning context, this study adds evidence on how interaction-related product features, psychological need satisfaction, perceived enjoyment, and continuance intention are associated in this category of educational technology.

Second, the study identifies exploratory differences in how the three psychological needs are associated with continuance intention within the estimated model. Autonomy and competence showed both direct associations and statistically significant indirect associations involving perceived enjoyment, whereas relatedness showed an indirect association but no significant direct association. Because these comparative differences were not specified a priori, they should be interpreted as empirically observed patterns rather than confirmatory evidence of distinct theoretical mechanisms. Nevertheless, the results suggest that the three needs need not exhibit comparable direct and indirect association patterns [14,18], providing a basis for more targeted confirmatory testing in future research.

Third, the study examines perceived enjoyment as the affective construct involved in the indirect associations between psychological needs and continuance intention. Within the S-O-R framework, the model estimates indirect association patterns involving need satisfaction, perceived enjoyment, and continuance intention, and shows that relatedness had the largest coefficient in relation to perceived enjoyment. These findings add evidence on enjoyment-related indirect association patterns in sustained engagement with socially interactive educational products [19–21], without implying temporal or causal mediation.

#### Practical implications.

The findings have several implications for the design and operation of AILDs.

First, response speed should not be treated as a substitute for relational interaction. Response timeliness showed associations with autonomy and competence but essentially no association with relatedness. Accordingly, improving response latency alone may not be sufficient when the design goal is to support users’ sense of being understood or accompanied. Relational cues such as warm language, patience, and supportive feedback may need to be considered separately.

Second, personalization should not automatically be equated with autonomy support. The non-significant association between perceived personalization and autonomy suggests that automatic adaptation may not necessarily give users a stronger sense of active choice or control. AILD interfaces could therefore consider making personalization more user-controllable, for example by allowing learners to adjust difficulty, choose among recommendations, view why content was recommended, or override system-generated learning plans. These design suggestions should be tested directly in future experimental research.

Third, perceived friendliness may be particularly relevant to relational experience. Its association with relatedness was among the comparatively larger feature–need associations in the model, although the corresponding effect size remained small. Warm language, encouraging feedback, and patient guidance may therefore be useful relational design directions, but their effects on sustained use require experimental or longitudinal confirmation.

Fourth, perceived enjoyment deserves attention because relatedness showed the only medium-sized effect in the model in its association with enjoyment, while enjoyment was positively associated with continuance intention. Product design may therefore benefit from considering enjoyable and relational interaction alongside functional performance, without assuming that these cross-sectional associations establish causal design effects.

### Limitations and future directions

This study has several limitations that also point to directions for future research.

First, the study uses cross-sectional data and cannot capture how users’ attitudes and behavior change over time. Because the central problem for AILDs is the decline of enthusiasm over time [8,9], future research could adopt a longitudinal design to track changes in psychological need satisfaction, enjoyment, and continuance intention across different stages of use. In particular, indirect associations estimated from cross-sectional data do not establish the temporal ordering implied by mediation and may differ from longitudinal mediation estimates [60,61]. The association patterns reported here should therefore be re-examined using longitudinal data.

Second, the sample was drawn from first-year senior high school students at a single public, science-and-technology-oriented high school in one Chinese city, which limits the generalizability of the findings. The characteristics of this population may also have shaped the observed association patterns. Students at this educational stage typically face substantial academic demands, which may make competence, learning efficiency, and timely feedback particularly salient in their evaluations of AI learning devices. This context may therefore have contributed to the comparatively stronger associations of response timeliness with autonomy and competence. At the same time, participants were approximately 15–16 years old, and their developmental stage may influence how autonomy and relatedness are experienced in interactions with AI-enabled learning technologies. The school’s science-and-technology orientation may also mean that students had greater familiarity with digital and AI-based learning tools than students in other educational settings. These possibilities cannot be tested directly with the present single-school sample and should therefore be treated as contextual interpretations rather than established explanations. Future research should replicate the model across schools with different academic profiles, grade levels, regions, and cultural contexts, and could also include parental perspectives to provide a broader account of AILD use in the home.

Third, the study measures continuance intention rather than actual use behavior. Although intention is an antecedent of behavior, a gap remains between intention and behavior. Future research could incorporate objective behavioral data such as device usage logs to further test the model’s predictive validity.

Fourth, the study focuses on four product features. Future research could include further potential stimulus variables, such as content quality, interface aesthetics, and price perception, and examine their relationships with the psychological constructs and association patterns examined here, in order to build a fuller theoretical framework of AILD user behavior.

Fifth, the model did not include perceived anthropomorphism as a construct. Although several stimulus features, particularly conversational style and friendly expression, carry anthropomorphic cues, future research could incorporate perceived anthropomorphism explicitly, for example as an antecedent of relational experience or as a moderator of feature-to-need associations, to clarify how human-likeness relates to the association patterns identified here.

Sixth, all focal constructs were measured by self-report from the same respondents within a single questionnaire session, creating a risk of common method bias. Although the procedural controls, Harman’s single-factor test, and full-collinearity assessment did not indicate an obvious severe problem, these diagnostics cannot establish the absence of common method variance. Shared method variance may have inflated some associations, particularly between conceptually and affectively proximate constructs such as relatedness and perceived enjoyment. Future studies could reduce this risk through temporal separation of predictors and outcomes, multi-source measurement, objective behavioral indicators, or the prospective inclusion of a theoretically unrelated marker variable.

## Conclusion

In response to the practical problem of AILDs, namely high enthusiasm shortly after purchase followed by the device being left idle after a period of use, this study moves beyond the conventional technology-acceptance perspective centered on cognitive and instrumental dimensions. It integrates the Stimulus-Organism-Response (S-O-R) framework with Self-Determination Theory (SDT) to examine an association model involving product features, psychological need satisfaction, perceived enjoyment, and continuance intention. Based on cross-sectional data from 726 first-year senior high school students in China, the main conclusions are as follows.

First, the feature–need associations were uneven rather than uniformly strong. Perceived friendliness with relatedness was among the comparatively larger feature–need associations (β=0.322, *f*^2^ = 0.085), whereas the expected associations of perceived personalization with autonomy (β=0.079, *p* = .071, *f*^2^ = 0.006) and response timeliness with relatedness (β=0.033, *p* = .530, *f*^2^ = 0.001) were not supported. These two unsupported associations are theoretically informative because they indicate that personalization should not automatically be equated with autonomy support and that response speed should not automatically be equated with relational connection.

Second, the three basic psychological needs showed differentiated association patterns with continuance intention. Autonomy and competence showed statistically significant direct and indirect associations with continuance intention, whereas relatedness showed a statistically significant indirect association involving perceived enjoyment but no significant direct association with continuance intention. These coefficient patterns indicate that the three needs need not exhibit comparable direct and indirect associations in the present sample, but they do not establish distinct causal or temporal pathways.

Third, perceived enjoyment occupied an important position in the estimated association structure. All three psychological needs showed statistically significant indirect associations with continuance intention involving perceived enjoyment; autonomy and competence additionally showed significant direct associations, whereas relatedness did not. Relatedness also showed the largest coefficient in relation to perceived enjoyment. For products such as AILDs, which combine functional and relational qualities, these findings suggest that enjoyment-related association patterns warrant further investigation, while their temporal and causal interpretation requires longitudinal or experimental evidence.

These conclusions carry implications for the design and operation of intelligent educational products. Because response timeliness was associated mainly with autonomy and competence rather than relatedness, functional performance such as response speed may need to be considered separately from relational qualities. The observed associations involving friendliness, relatedness, enjoyment, and continuance intention further suggest that relational and enjoyable interaction deserves attention alongside functional efficiency. These design implications remain provisional because the present study does not establish causal effects. Constrained by cross-sectional data, a single-grade sample, and the measurement of intention rather than behavior, the findings should be further examined through longitudinal tracking, replication across broader and more diverse samples, experimental manipulation, and objective behavioral data. The differentiated direct and indirect association patterns identified here provide an empirical basis for subsequent research on sustained engagement with socially interactive AI-enabled educational products.
